# Crystallographic studies of gas sorption in metal–organic frameworks

**DOI:** 10.1107/S2052520614009834

**Published:** 2014-05-24

**Authors:** Elliot J. Carrington, Iñigo J. Vitórica-Yrezábal, Lee Brammer

**Affiliations:** a Department of Chemistry, University of Sheffield, Brook Hill, Sheffield S3 7HF, England

**Keywords:** metal–organic frameworks, gas sorption, framework flexibility

## Abstract

Metal–organic frameworks (MOFs) are a class of porous crystalline materials of modular design. One of the primary applications of these materials is in the adsorption and separation of gases, with potential benefits to the energy, transport and medical sectors. *In situ* crystallography of MOFs under gas atmospheres has enabled the behaviour of the frameworks under gas loading to be investigated and has established the precise location of adsorbed gas molecules in a significant number of MOFs. This article reviews progress in such crystallographic studies, which has taken place over the past decade, but has its origins in earlier studies of zeolites, clathrates *etc*. The review considers studies by single-crystal or powder diffraction using either X-rays or neutrons. Features of MOFs that strongly affect gas sorption behaviour are discussed in the context of *in situ* crystallographic studies, specifically framework flexibility, and the presence of (organic) functional groups and unsaturated (open) metal sites within pores that can form specific interactions with gas molecules.

## Abstract 2

Adsorption and separation of gases is one of the primary applications of the class of materials known as metal–organic frameworks (MOFs). The role of crystallography in characterizing adsorbed gas molecules and changes in framework structure upon gas sorption is reviewed.

## 1. Introduction   

Metal–organic frameworks (MOFs), also known as porous coordination polymers (PCPs), are a class of materials comprising metal ions or small metal clusters linked through coordination bonds *via* organic ligands into two-dimensional or three-dimensional periodic assemblies. This class of materials is the subject of extensive and growing worldwide research activity. The materials have been targeted for a variety of applications due to their high porosity, large surface areas, structural diversity, and both geometric and chemical tunability (Zhou *et al.*, 2012 ▶). Prominent applications include gas storage, gaseous and other molecular separations, use as chemical sensors, and in light harvesting, biomedicine and catalysis. Each application area has been the subject of review articles (Horcajada *et al.*, 2012 ▶; Kreno *et al.*, 2012 ▶; Li *et al.*, 2012 ▶; Liu *et al.*, 2012 ▶; Sumida *et al.*, 2012 ▶; Wang *et al.*, 2012 ▶; Yoon *et al.*, 2012 ▶).

The gas sorption properties of MOFs for use in storage and separation applications are considered particularly important and are frequently studied, both for new and existing MOFs (Lin *et al.*, 2007 ▶; Collins & Zhou, 2007 ▶; Dincă & Long, 2008 ▶; Murray *et al.*, 2009 ▶; Li *et al.*, 2009 ▶; D’Alessandro *et al.*, 2010 ▶; Sumida *et al.*, 2012 ▶; Suh *et al.*, 2012 ▶; Wu *et al.*, 2012 ▶). Experimental studies typically involve gravimetric or volumetric adsorption measurements. These measurements allow the behaviour of the framework to be assessed and, in particular, enable both the amount of gas adsorbed at specific pressures and the overall maximum uptake achievable to be determined. Gravimetric or volumetric adsorption measurements, however, do not provide information on the location of gas molecules retained within the framework, and thereby yield only limited information on the mechanism for adsorption.

Knowledge of the sites of gas adsorption within the framework permits an understanding of the interactions occurring upon adsorption and could ultimately lead to the design of improved framework materials for gas adsorption and separation applications. Information on the binding modes of gases within MOFs has been obtained by several different methods, for example, inelastic neutron scattering (INS) for H_2_ adsorption in [Zn_4_(μ_4_-O)(BDC)_3_] (MOF-5) (Rosi *et al.*, 2003 ▶), Raman spectroscopy for CO_2_ adsorption in [Zn(MeIm)_2_] (ZIF-8) and [Zn(SiF_6_)(pyz)_2_] (Kanoo *et al.*, 2012 ▶; Kumari *et al.*, 2013 ▶), IR spectroscopy studies for CO and CO_2_ in [Zr_6_(μ_4_-O)(μ_4_-OH)(BDC)_6_] (UiO-66) (Wiersum *et al.*, 2011 ▶) and NO in [Fe_3_(μ_3_-O)(OH)(BDC)_3_] (MIL-88B(Fe)) (McKinlay *et al.*, 2013 ▶), solid-state NMR spectroscopy for CO and CO_2_ adsorption in [Cu_3_(BTC)_2_] (HKUST-1) (Gul-E-Noor *et al.*, 2013 ▶). Quantum chemical calculations have also been employed to investigate H_2_ adsorption (Han *et al.*, 2009 ▶) and to characterize the mechanism involved in CO_2_ adsorption in the amine-functionalized MOF mmen-Mg_2_(dobpdc) (dobpdc^4−^ = 4,4′-dioxido-biphenyl-3,3′-dicarboxylate; mmen = *N*,*N*′-dimethylethylenediamine; Planas *et al.*, 2013 ▶). However, as with other areas of solid-state structural elucidation, crystallographic methods have the potential to provide the most definitive structural information.

There are many examples of crystallographic characterization of molecular guests in MOFs, in particular solvent molecules which are present in the as-synthesized materials, but such characterization is often difficult due to disorder and/or partial occupancies of the guests. Such challenges also commonly arise when determining the location of trapped gas molecules in MOFs.

Trapped gas molecules have been identified and characterized crystallographically in other porous materials prior to the development of MOFs in the mid-1990s. Work on zeolites is of particular relevance due to their similar properties to MOFs. Early examples are studies by Riley and Seff on adsorption of acetylene and carbon monoxide in various metal exchanged zeolites (Amaro & Seff, 1973 ▶; Riley & Seff, 1973 ▶, 1974 ▶; Riley *et al.*, 1975 ▶). More recent studies of zeolites include location of noble gas atoms (Wright *et al.*, 1984 ▶; Cho *et al.*, 2012 ▶) and of CO_2_ molecules (Lozinska *et al.*, 2012 ▶; Wong-Ng *et al.*, 2013 ▶). All are studies by X-ray or neutron powder diffraction. Similar studies of other porous inorganic materials have also been reported, for example, N_2_ and H_2_ adsorption in a magnesium borohydride material (Filinchuk *et al.*, 2011 ▶). Crystallographic studies on the gas hydrates have also illustrated the possibility of characterizing, by single-crystal X-ray and neutron diffraction and by powder diffraction, a variety of small gas molecules within hydrogen-bonded water clathrate cages that are templated by the included gases (Jeffrey & McMullan, 1967 ▶; Pauling & Marsh, 1952 ▶; McMullan & Kvick, 1990 ▶; Mahajan *et al.*, 2000 ▶; Hoshikawa *et al.*, 2006 ▶; Chakoumakos *et al.*, 2011 ▶).

Further developments in non-ambient crystallography and, in particular, the development of crystallographic capabilities at synchrotrons (Brunelli & Fitch, 2003 ▶; Takata, 2008 ▶; Thompson *et al.*, 2009 ▶; Jensen *et al.*, 2010 ▶; Nowell *et al.*, 2012 ▶; Hill, 2013 ▶) have enabled *in situ* gas sorption studies to be carried out on MOFs, which, due to their high porosity, often diffract quite weakly. This review focuses on such studies of MOFs that involve crystallographic location of the entrapped gas molecules. Selected examples are discussed. A more extensive list of such studies is provided in Table 1 ▶ and a list of abbreviations used in this review is included in the *Appendix* [App appa].

## 2. Early examples   

**Figure 1:**
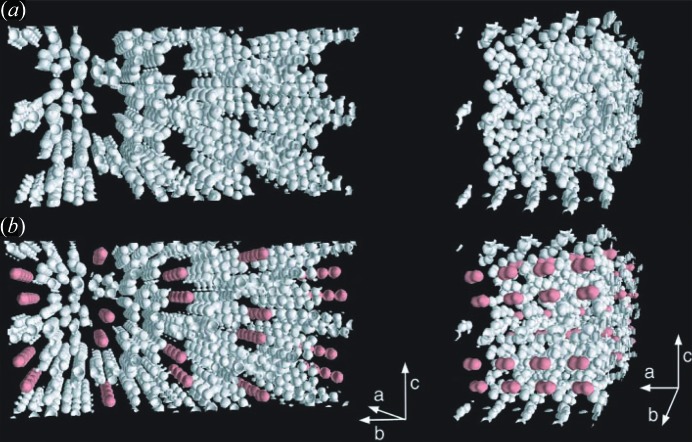
MEM electron densities of (*a*) anhydrous CPL-1 without O_2_ molecules at 120 K and (*b*) CPL-1 with adsorbed O_2_ at 90 K as an equal-density contour surface. The equicontour level is 1.0 e Å^−3^. (Reproduced from Kitaura *et al.*, 2002 ▶, with permission from the AAAS.)

**Figure 2:**
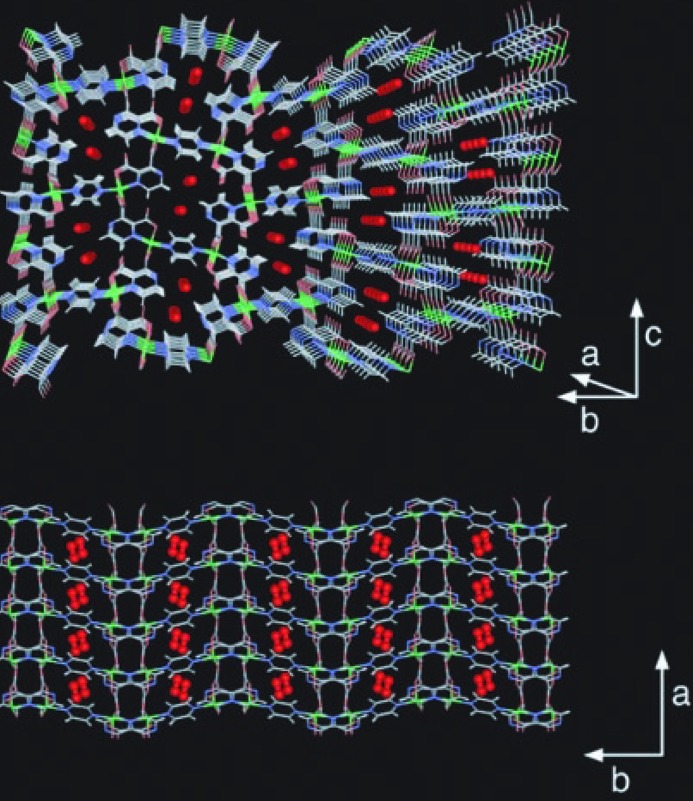
Crystal structure of CPL-1 with adsorbed O_2_ at 90 K. View down the *a*-axis (top). View down the *c*-axis (bottom). (Reproduced from Kitaura *et al.*, 2002 ▶, with permission from the AAAS.)

The possibility of gas sorption in MOFs was recognized very early in the development of the field (Kondo *et al.*, 1997 ▶; Li *et al.*, 1998 ▶, 1999 ▶; Chui *et al.*, 1999 ▶; Eddaoudi *et al.*, 2000 ▶, 2002 ▶; Fletcher *et al.*, 2001 ▶; Barthelet *et al.*, 2002 ▶). The first *in situ* studies by crystallographic methods of gas sorption in MOFs followed soon after. In 2002, Kitagawa, Takata and coworkers used synchrotron X-ray powder diffraction to examine physisorbed O_2_ in the channels of [Cu(pzdc)(pyz)], CPL-1 (pzdc = 2,3-pyrazinedicarboxylate; pyz = pyrazine; Kitaura *et al.*, 2002 ▶). The study used the maximum entropy method (MEM)/Rietveld methodology developed by Takata (Takata *et al.*, 1995 ▶, 2001 ▶; Tanaka *et al.* 2002 ▶) to produce a precise electron-density map from high-resolution X-ray powder diffraction (PXRD) patterns (Takata, 2008 ▶) collected under an O_2_ atmosphere. The data were recorded using an O_2_ pressure of 80 kPa (0.8 bar) and temperatures from 90 to 300 K at the SPring-8 synchrotron. Initial removal of guest water molecules was carried out by heating under reduced pressure with no change in the framework structure. The electron density map generated from the MEM analysis was then used to identify the O_2_ sites within the pores (at 90 K; Fig. 1 ▶). Final Rietveld refinement yielded indices of fit *R*_wp_ = 0.021 and *R*_I_ = 0.039. The oxygen sites were observed as peanut-shaped electron densities in the middle of the channels, accounting for 15.8 (1) electrons based on the MEM analysis and suggesting diatomic oxygen. A ratio of one O_2_ molecule per Cu atom was observed, corresponding well to the adsorption isotherms recorded at 77 K, which suggested a saturation of one O_2_ per Cu atom. The ordering of the O_2_ molecules indicated that they more closely resembled O_2_ in the solid state rather than the liquid or gaseous state despite the experimental conditions (*T* > b.p. of O_2_), suggesting a significant confinement effect. Furthermore, the intermolecular distance between pairs of O_2_ molecules [3.28 (4) Å] was observed to be lower than the minimum of the Lennard–Jones potential (3.9 Å) and suggests the formation of van der Waals dimers (Fig. 2 ▶). The dimers align along the *a*-axis to form a one-dimensional ladder structure, leading to antiferromagnetic coupling as confirmed by magnetic susceptibility measurements.

**Figure 3:**
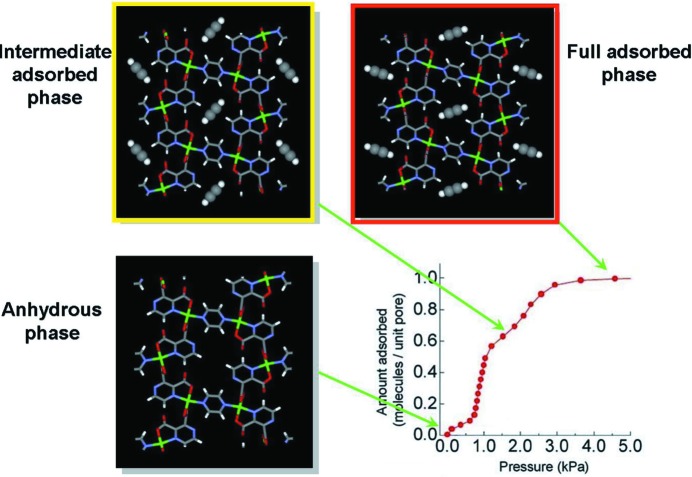
Crystal structure of CPL-1 viewed along the *a*-axis, in its evacuated form, and with channels partially filled (intermediate phase) and filled (full adsorbed phase), showing changes in acetylene-to-framework C—H⋯O hydrogen bonding on increasing gas loading. (Reproduced from Takata, 2008 ▶, with permission from the IUCr.)

Further crystallographic studies have been conducted of CPL-1 under atmospheres of a number of gases, including N_2_, Ar, CH_4_, H_2_ and C_2_H_2_ (Kitaura *et al.*, 2005 ▶; Kubota *et al.*, 2005 ▶, 2006 ▶, 2007 ▶; Matsuda *et al.*, 2005 ▶). The nitrogen uptake showed similar results to oxygen, forming van der Waals dimers in one-dimensional arrays, but the accessible pore surface for both the Ar and CH_4_-filled frameworks was seen to be significantly different to those of the oxygen and nitrogen-containing versions. This was suggested to result from framework flexibility and considered to be an effect of induced fitting *via* molecule-to-pore surface interactions. The confinement of acetylene (C_2_H_2_) within CPL-1 was even more interesting. The structure at different acetylene loadings was shown to have an intermediate and a saturated phase, which exhibit different acetylene-to-framework interactions (Fig. 3 ▶; Matsuda *et al.*, 2005 ▶). At lower loadings, a meta-stable phase formed with an interaction between the acetylene and the two metal-coordinated carboxylate O atoms, but under saturated conditions a slight rotation of the acetylene molecules occurs, which aligns them with two uncoordinated O atoms of the carboxylates to form stronger C—H⋯O hydrogen bonds. Subsequently, the pyrazine rings of the framework are then seen to rotate. Contractions are observed in the unit cell on going from the intermediate phase to the acetylene-saturated phase, which contains acetylene at storage densities 200 times the compression limit of free acetylene. These specific guest-framework interactions provide a clear rationale for the greatly enhanced adsorption of C_2_H_2_ over CO_2_, particularly at low pressures. Analogous studies on hydrogen adsorption also suggest interactions with carboxylate O atoms. Although H atoms are not clearly resolved, an occupancy of 0.3H_2_ per site is recovered from the MEM/Rietveld analysis (Kubota *et al.*, 2005 ▶), consistent with an estimate of adsorbed H_2_.

**Figure 4:**
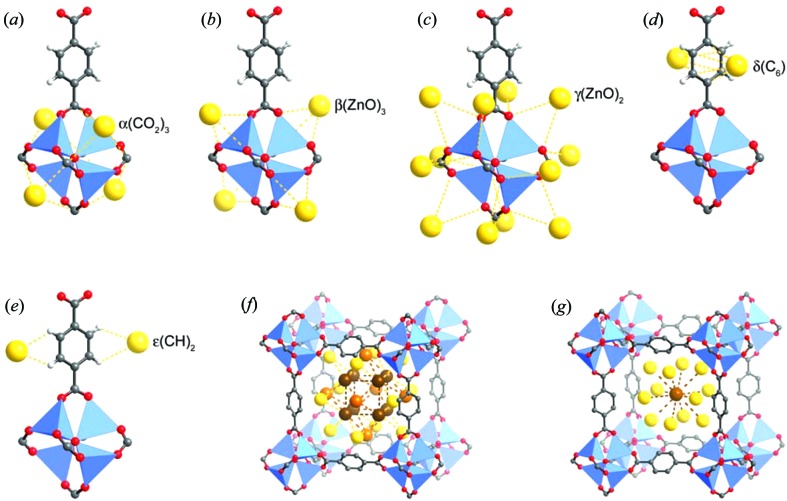
Eight symmetry-independent adsorption sites in MOF-5, each partially occupied by Ar atoms, as identified by single-crystal X-ray diffraction at 30 K. Sites α–∊ shown in (*a*)–(*e*) are in close proximity to framework atoms. Nitrogen molecules only populate sites α, γ and ∊ at 30 K, but instead populate sites β and δ alongside α at higher temperatures. (Reproduced from Rowsell, Spencer *et al.*, 2005 ▶, with permission from the AAAS.)

In 2005, Yaghi, Howard and coworkers reported single-crystal X-ray diffraction studies of adsorption experiments of Ar and N_2_ in MOF-5 (Rowsell, Spencer *et al.*, 2005 ▶), a primitive cubic framework, comprising Zn_4_(μ_4_-O)^6+^ clusters linked *via* terephthalate ligands. Single-crystal X-ray diffraction had been previously used to determine the positions of gas molecules, specifically CO_2_, in a dynamically porous one-dimensional coordination polymer under a sealed gaseous atmosphere (Takamizawa *et al.*, 2003 ▶), but not on a three-dimensional framework with such large voids and with the potential for many crystallographically independent adsorption sites. The study indicated eight different adsorption sites: five close to the framework and an additional three forming a second layer within the pores of the MOF (Fig. 4 ▶). All the sites exhibited partial occupancies and seemed to be intrinsic to the framework, with the same sites being observed in both the N_2_ and Ar adsorption cases, albeit with different relative populations. Diffraction studies were conducted on a laboratory diffractometer at temperatures from 293 to 30 K. By controlling the temperature and looking at the relative occupancies the authors were able to rank the preferential adsorption sites. Gas molecules could not be located at 293 K, but electron density attributable to the gas molecules was clearly evident at 30 K, which is below the freezing point of the gases. The most populated site for both gases (site α) was very close to the metal cluster, with interactions involving three carboxylate groups and three Zn atoms. Other binding sites, such as interactions with the edge of the aromatic ring (sites δ and ∊; see *d* and *e* in Fig. 4 ▶), were previously unobserved in gas sorption studies in the presence of aromatic moieties.

Over the last decade, crystallographic characterization of gas-containing MOFs has progressed and a number of different studies have been reported. This review will focus on selected examples to illustrate different aspects of the field. A more complete list of studies is provided in Table 1 ▶. We will begin with a survey of some examples that illustrate the different crystallographic techniques used. The review will then consider situations where the unique properties of the framework, such as its flexibility, or the presence of functional groups or open metal sites have been shown to influence the location of the gas sorption sites.

## 3. X-ray *versus* neutron diffraction   

Single-crystal X-ray diffraction has been the main method for definitive structural characterization over the last century and has recently been used effectively to locate gas molecules entrapped in the pores of MOFs in several studies similar to those by Yaghi, Howard and co-workers (see above). These *in situ* diffraction experiments typically adopt one of two approaches, in both of which the crystal is situated inside a glass capillary. A simpler experimental design involves the capillary being filled with the desired gas to a specified pressure and sealed to maintain the atmosphere, enabling measurement at a single pressure. A change in temperature can be used to change the relative pressure (*p*/*p*_0_). Alternatively, and now more commonly, the capillary is connected *via* tubing to a gas manifold that enables evacuation and then dosing the capillary with gas to a desired pressure. The latter arrangement has the advantage of enabling a sequence of measurements to be made at different pressures at the same temperature (as well as varying temperature), and for measurements involving several gases to be made on the same crystal. Such experimental set-ups, known as static pressure cells, are now found at a number of beamlines at synchrotron facilities worldwide. A further alternative is a flow cell, in which the gas is continuously flowed over the crystalline sample within an environmental cell during the diffraction experiment.

**Figure 5:**
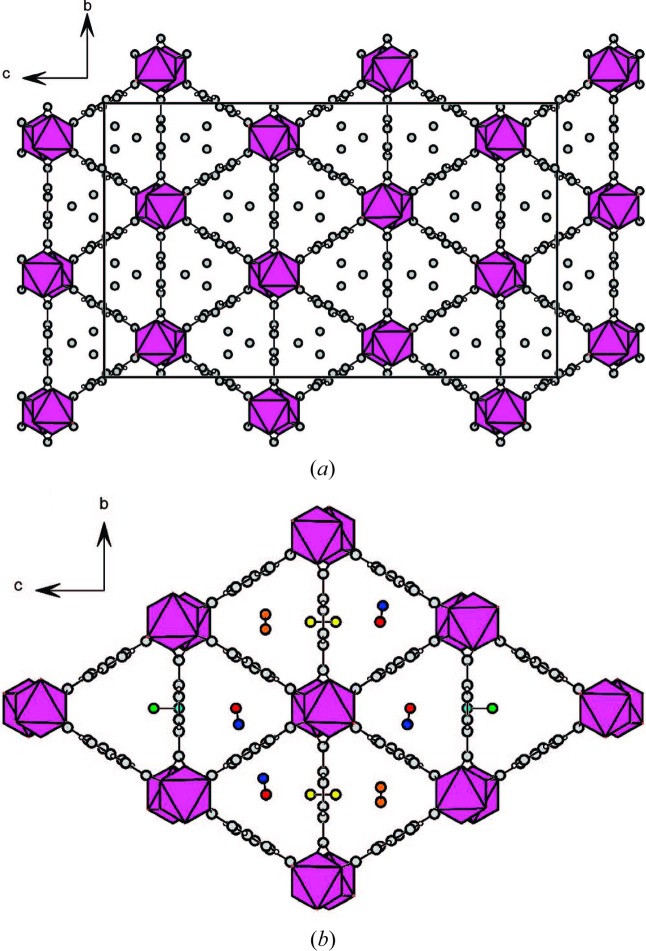
Location of (*a*) adsorbed CH_4_ (9 bar) and (*b*) adsorbed C_2_H_6_ (5 bar) in the channels of the MOF [Sc(BDC)]. Disorder of CH_4_ molecules is shown. One of three locations in the disordered model for C_2_H_6_ is shown. H atoms are not shown. (Reproduced from Miller *et al.*, 2009 ▶, with permission from the American Chemical Society.)

**Figure 6:**
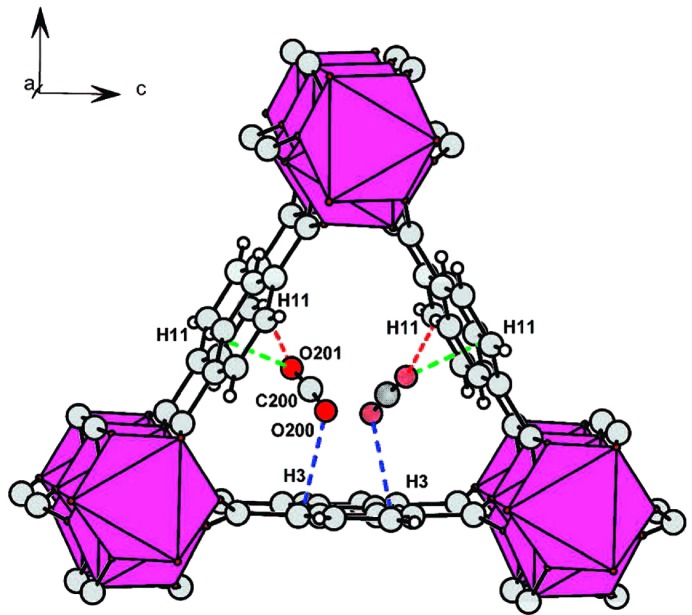
One of two inequivalent channels in [Sc(BDC)] following adsorption of CO_2_ (1 bar). CO_2_ molecules are disordered over the two sites (as shown) in this channel. (Reproduced from Miller *et al.*, 2009 ▶, with permission from the American Chemical Society.)

An illustrative example of a static cell study is that by Miller *et al.* carried out at the Swiss–Norwegian beamline (BM01) at ESRF (Miller *et al.*, 2009 ▶). The study examined gas adsorption sites in a Sc(BDC) MOF (BDC = 1,4-benzenedicarboxylate or terephthalate), which is a small-pore MOF with one-dimensional channels and a poor affinity for water. Single crystals were mounted on a glass fibre and glued inside a 0.3 mm quartz capillary. The capillary was evacuated and the diffraction pattern checked under vacuum, before separate introduction of a sequence of gases. The gases studied were CO_2_, H_2_, CH_4_ and C_2_H_6_, each at a single gas pressure and temperature selected based on previously measured adsorption isotherms and chosen to ensure sufficient gas uptake to enable crystallographic detection of the gas molecules. The diffraction experiment was performed at 0.25 bar and 80 K for H_2_, 1 bar and 235 K for CO_2_, and at 230 K for CH_4_ (9 bar) and C_2_H_6_ (5 bar). The CO_2_ molecules and the C atoms of adsorbed CH_4_ and C_2_H_6_ molecules could be modelled in the pores of the MOF. The hydrocarbon gas uptake resulted in no change to the framework structure or the space group of the crystals (Fig. 5 ▶). Introduction of CO_2_ (Fig. 6 ▶) or H_2_, by contrast, resulted in a lowering of the symmetry from orthorhombic *Fddd* to monoclinic *C*2/*c*. This change arises due to rotation of the terephthalate groups upon adsorption of CO_2_ or H_2_, leading to adjacent channels becoming inequivalent. In the case of CO_2_, weak C—H⋯O interactions (H⋯O 2.87–2.98 Å) involving phenyl H atoms are observed. Calorimetric studies indicate that binding to CO_2_ is the strongest of the four gases, but still of modest strength (Δ*H*_ads_ = −20 kJ mol^−1^ at room temperature). Thus, the authors note that it is the small size of the channels that enables crystallographic location of the physisorbed gas molecules even at 230 K.

Although X-ray crystallographic studies of gas adsorption in MOFs have primarily involved the use of synchrotron facilities, laboratory diffractometers have also been used (Takamizawa *et al.*, 2003 ▶; Rowsell, Spencer *et al.*, 2005 ▶). One such example is the work by Zhang and Chen on the absorption of N_2_, CO_2_ and C_2_H_2_ in a metal azolate framework, [Cu(etz)]_*n*_ (MAF-2, Hetz = 3,5-diethyl-1,2,4-triazole; Zhang & Chen, 2009 ▶). Single crystals of MAF-2 were sealed inside glass capillaries together with the desired gas (C_2_H_2_ or CO_2_). X-ray data were collected at temperatures of 123 and 293 K for crystals with maximum loading (MAF-2·C_2_H_2_ and MAF-2·CO_2_). The position and anisotropic displacement parameters of the entrapped acetylene molecules could be refined without restraints, using the 123 K data, consistent with strong localization of the acetylene molecules. In contrast, entrapped CO_2_ molecules could only be modelled using crystallographic restraints. The acetylene-containing crystal structure could also be modelled at room temperature, whereas at this temperature CO_2_ within the pores could not. The crystallographic results are consistent with gravimetric gas adsorption measurements, which suggested a preferential uptake of C_2_H_2_ over CO_2_ (Zhang & Chen, 2009 ▶). Although the space group was maintained upon adsorption of the two gases, the crystallographic models showed slight deformations of the framework structure even at very low loadings of gas.

**Figure 7:**
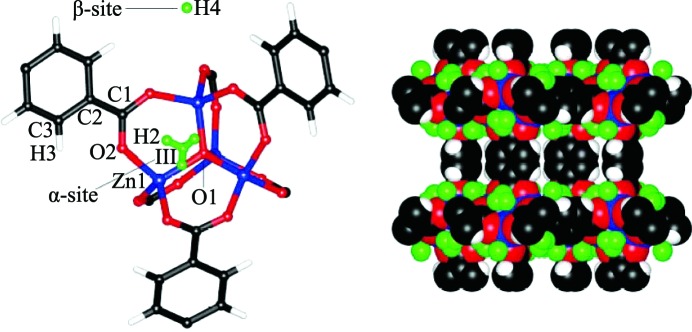
The two H_2_ sites (α and β) identified by single-crystal neutron diffraction in MOF-5, shown for a single framework node (left) and for a section of the framework (right). (Reproduced from Spencer *et al.*, 2006 ▶, http://dx.doi.org/10.1039/B511941C, with permission from the Royal Society of Chemistry ).

Accurate location of H atoms of the guest molecules is typically not possible using X-ray diffraction, since scattering intensity is related to electron density. The situation is particularly problematic when considering the location of H_2_ molecules in the pores of MOFs. Most studies involving H_2_ molecule location within MOFs therefore typically use neutron diffraction instead, for which the scattering length for hydrogen is more comparable to that of other elements. Two such studies using different neutron diffraction techniques to identify the hydrogen adsorption sites in MOF-5 were reported in 2005–2006 (Yildirim & Hartman, 2005 ▶; Spencer *et al.*, 2006 ▶). Yaghi, Howard and co-workers extended their earlier single-crystal X-ray diffraction studies by using single-crystal neutron diffraction and Yildirim and Hartman used neutron powder diffraction (NPD). The single-crystal diffraction data were recorded at various temperatures and identified two different sites for hydrogen adsorption (Fig. 7 ▶). These directly correspond, respectively, to the α and β sites from the previous X-ray study using argon and nitrogen (Figs. 4 ▶ *a* and *b*). Location of H_2_ at the α site could be modelled at 50 K or below, including modelling of atomic positions, whereas population of the β site could only be identified at 5 K, but with individual atoms unable to be modelled. This indicates that the molecules are not highly localized, particularly at higher temperatures. The work only partially agreed with previous inelastic neutron scattering (INS) data (Rosi *et al.*, 2003 ▶; Rowsell, Eckert & Yaghi, 2005 ▶), which suggest that the hydrogen did indeed interact with the α site, but also with the γ site identified in the previous X-ray study of Ar and N_2_ uptake (Rowsell, Spencer *et al.*, 2005 ▶), although not with the β site identified in the neutron diffraction study (Spencer *et al.*, 2006 ▶).

The use of hydrogen in neutron diffraction experiments can present difficulties due to its large incoherent scattering cross-section, a problem that is more challenging for powder diffraction than single-crystal diffraction experiments. Such a problem does not arise for deuterium, which exhibits much smaller incoherent scattering, as well as a larger (coherent) scattering length. Therefore, replacement of hydrogen by deuterium, where possible, in the materials studied is a common experimental approach in neutron diffraction. Considering gas sorption by MOFs, this can involve MOFs comprising perdeuterated ligands or the use of D_2_ instead of H_2_ (or, more generally, the use of deuterated gases; Peterson *et al.*, 2006 ▶; Wu, Simmons, Liu *et al.*, 2010 ▶). The NPD study by Yildirim and Hartman was conducted at 3.5 K using a perdeuterated MOF-5 sample loaded with D_2_ and the structure refined by Rietveld methods (Yildirim & Hartman, 2005 ▶). Analyses were carried out for several gas loadings ranging from 4D_2_ molecules per Zn centre to 46D_2_ per Zn. Four different sites were identified for the location of D_2_ molecules. These sites correspond to sites α–δ from the single-crystal X-ray study (of Ar and N_2_) and the first two relate directly to the two sites identified in the single-crystal neutron study. One of the other two sites (γ) therefore also matches the secondary site suggested by the INS study (Rowsell, Eckert & Yaghi, 2005 ▶). It is not clear if the increased number of binding sites observed in this study is due to the lower temperature or the deuteration (of framework and gas). Although there are some differences, this series of studies by different groups has reached similar conclusions regarding the preferred binding sites.

In further studies by Yildrium and co-workers, NPD data were recorded at 3.5 K with various D_2_:Zn ratios for the MOF [Zn(mIm)_2_]_*n*_ (ZIF-8; ZIF = zeolitic imidazolate framework; HmIm = 2-methylimidazole). Six different D_2_ sites were identified after Rietveld refinement of the structural model (Wu *et al.*, 2007 ▶). The principal site, designated based on the relative D_2_ occupancies, involves interaction with the imidazolate linker and contrasts with the previous examples discussed where the main binding mode was to the metal cluster.

**Figure 8:**
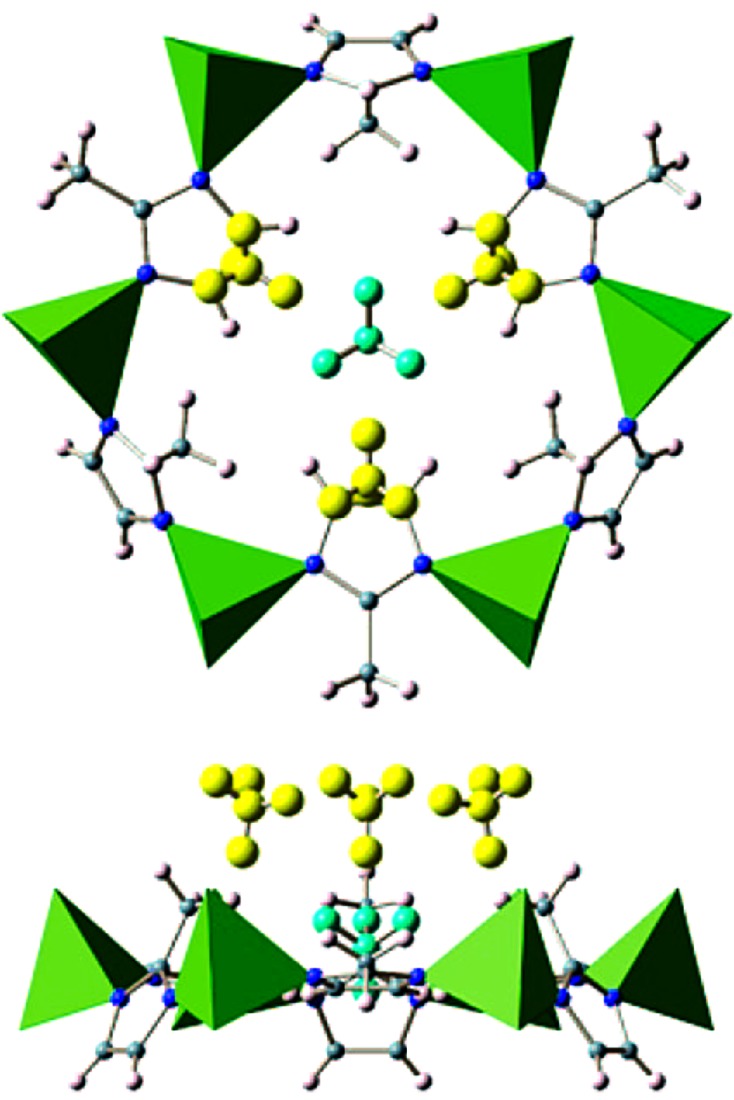
Primary (yellow) and secondary (cyan) sites for methane (CD_4_) in ZIF-8 shown in two perpendicular views of the hexagonal pore. (Reproduced from Wu *et al.*, 2009 ▶, with permission from the American Chemical Society).

*In situ* NPD has also been used in studies of adsorption of gases other than hydrogen. Wu *et al.* used Rietveld refinements to determine CD_4_ locations ZIF-8 and MOF-5 (Wu *et al.*, 2009 ▶). Upon low loadings of gas molecules both frameworks were shown to adsorb methane at the primary binding sites discussed in the previous studies (two sites for ZIF-8 and one site for MOF-5). Population of these binding sites resulted in no change in the crystalline phase of the framework upon cooling to 3.5 K and gave defined CD_4_ orientations which fitted with the symmetry of the space group (Fig. 8 ▶). Higher loadings at 80 K did not show any additional well defined CD_4_ molecules, but the primary binding sites remained occupied. Cooling these samples below 60 K resulted in a reversible change in crystalline phase and the crystal structures had to be solved in a lower symmetry supercell. This was attributed to slight deformations in the framework caused by intermolecular repulsions from the confined methane (Wu *et al.*, 2009 ▶).

There are several reports in which useful information about the behaviour of MOFs has been found from the *in situ* powder diffraction studies of gas-loaded samples, without the use of Rietveld refinements to determine the location of the gas molecules. These include the use of *in situ* X-ray powder diffraction to examine phase transitions in [Zn(TCNQ-TCNQ)(bpy)] occurring upon the selective adsorption of O_2_ and NO gas (Shimomura *et al.*, 2010 ▶), investigation of structural changes in DMOF, [Zn_2_(BDC)_2_(DABCO)], upon co-adsorption of CO_2_ and fluorescent guest molecules (Yanai *et al.*, 2011 ▶), and studies of framework flexibility and reversibility upon CO_2_ adsorption above and below its triple point (Yang, Lin *et al.*, 2012 ▶).

## 4. Flexible MOFs   

Guest-responsive behaviour in some MOFs, for example the ability to change pore size or framework structure upon the introduction of various gases or other guest molecules, may potentially lead to important applications in gas sorption and separation. The effects are often apparent from the shape of adsorption isotherms, but can also be followed by crystallographic methods. Such processes usually involve a change of the unit-cell dimensions which is identifiable by the shifts of the Bragg peaks in the powder diffraction pattern. Changes in the framework structure can be reversible upon desorption of the gas and such behaviour is often termed ‘breathing’.

**Figure 9:**
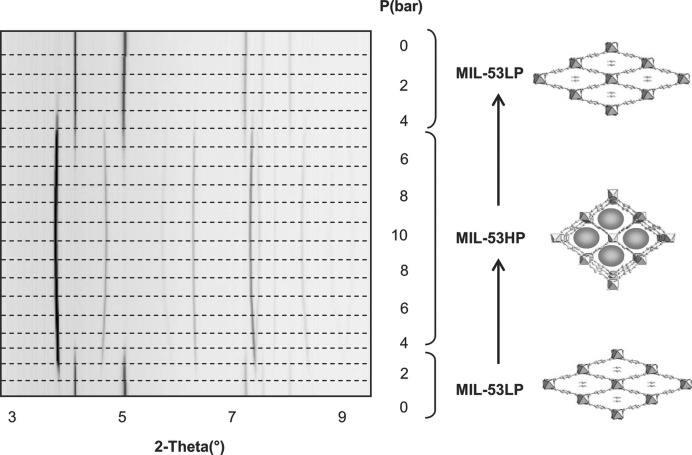
‘Breathing’ effect of MIL-53(Cr) at different pressures of CO_2_, demonstrated by *in situ* X-ray powder diffraction and the corresponding structural changes (LP = low pressure; HP = high pressure). (Reproduced from Serre *et al.*, 2007 ▶, with permission from Wiley.)

The most-studied MOF family that exhibits ‘breathing’ behaviour is probably the MIL-53(M) series. These isostructural MOFs involve trivalent metal ions (*M* ^3+^) coordinated to terephthalate (BDC) linkers and adopt large or narrow pore structures depending on the absorption of particular guests (Hamon *et al.*, 2009 ▶). In 2006 Llewellyn *et al.* reported on the framework breathing of hydrated MIL-53(Cr) and MIL-53(Al) on the introduction of CO_2_ gas in a study by *in situ* powder X-ray diffraction (Llewellyn *et al.*, 2006 ▶). In a more detailed follow-up study in 2007, Serre and Férey examined anhydrous MIL-53(Cr) upon addition of CO_2_ between 0 and 10 bar, and studied the response of the framework *in situ* by synchrotron XRPD (Fig. 9 ▶). On initial adsorption (up to 4 bar) the framework adopts the low pressure (narrow pore) form (Serre *et al.*, 2007 ▶). Upon higher loadings of gas (above 5 bar) the framework expands to give the high-pressure (large pore) form. Upon slowly removing the CO_2_ pressure the large pore phase is retained down to 2 bar, before fully converting to the narrow pore version again, showing a hysteresis that correlates with the corresponding adsorption isotherms. The framework also showed a reversible cycling effect upon removal and re-dosing of CO_2_, which could be important for future gas storage/separation applications.

The study also shows that, upon degassing, the MIL-53(Cr) framework exists in the large pore form, and the addition of a small amount of CO_2_ causes the framework to close due to host–guest interactions. To study this behaviour, Rietveld refinements on a powder pattern measured at below 1 bar CO_2_ were used in conjunction with periodic DFT calculations and *in situ* IR spectroscopy. An electron donor–acceptor interaction between the hydroxyl O atom of the framework and the adsorbed CO_2_ is indicated to be responsible for the breathing effect (Serre *et al.*, 2007 ▶).

**Figure 10:**
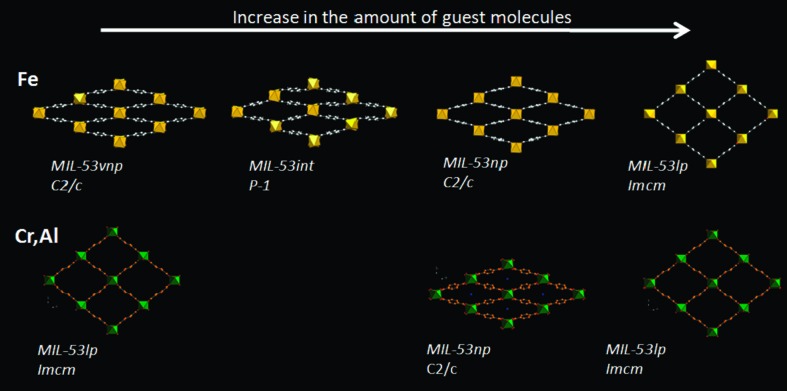
Structural evolution of the various MIL-53 series analogues with increasing amount of alkane guest. Top MIL-53(Fe), bottom (MIL-53(Cr,Al). (Reproduced from Llewellyn *et al.*, 2009 ▶, with permission from the American Chemical Society.)

**Figure 11:**
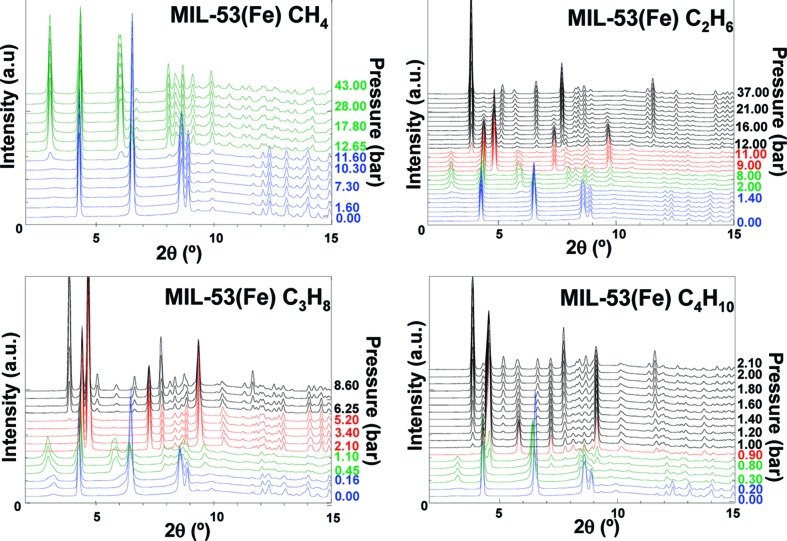
*In situ* XRPD studies of hydrocarbon adsorption in MIL-53(Fe). Phase changes upon increasing pressure are indicated by colour changes in patterns (blue = vnp; green = intermediate; red = np; black = lp). (Reproduced from Llewellyn *et al.*, 2009 ▶, with permission from the American Chemical Society.)

The differences between the chromium and iron analogues of the MOF, MIL-53(Cr) and MIL-53(Fe), under the absorption of light gaseous hydrocarbons have also been explored by powder X-ray diffraction (Llewellyn *et al.*, 2008 ▶, 2009 ▶; Rosenbach *et al.*, 2010 ▶). MIL-53(Fe) showed much more complex behaviour than its sister compounds with a multi-step breathing process (Fig. 10 ▶). A total of four different pore-opening stages were reported upon increasing gas pressure, starting with a very narrow pore (vnp) phase (*C*2/*c*), then moving to an intermediate phase (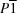), next to the narrow pore phase analogous to the Cr and Al frameworks (*C*2/*c*) and finally the large pore form (*Imcm*) at the highest pressure. In addition, the relative pressures required to convert from one phase to another were shown to greatly vary based on the hydrocarbon chain length. Introduction of CH_4_ caused the structure to change to the intermediate phase at around 12 bar pressure, but then gave no further structural changes, whereas uptake of C_4_H_10_ caused the framework to go through all four phases and be fully converted to the large pore form before reaching 2 bar. C_2_H_6_ and C_3_H_8_ showed intermediate activity, confirming a trend in behaviour of the framework that correlates with size of the alkane gas molecules (Llewellyn *et al.*, 2009 ▶; Fig. 11 ▶). A similar trend is observed in the pressure at which the narrow-to-large pore transitions occur in MIL-53(Cr) (Llewellyn *et al.*, 2008 ▶). MIL-53(Fe) was also shown to exist in the closed (very narrow pore) form when evacuated in contrast to the Cr (and Al) analogue which require the absorption of a small amount of guest for it to close (Llewellyn *et al.*, 2009 ▶).

The effect of mixed gases on the breathing behaviour of MIL-53(Cr) has also been crystallographically explored using co-adsorption of CO_2_ and CH_4_. It was found that mixtures with equimolar or high levels of CO_2_ followed the normal breathing behaviour, where the pores initially closed at low gas pressures and then reopened at higher pressures. However, mixtures high in CH_4_ did not show the breathing behaviour and constantly remained in the large pore form, similar to that observed for adsorption of pure methane. Control of the breathing behaviour, therefore, was attributed to the partial pressure of CO_2_, with the narrow pore form thought to exclude CH_4_ and contain mainly CO_2_. This could potentially offer high selectivity for separation of these two gases (Hamon *et al.*, 2009 ▶).

The changes that occur when terephthalate ligands containing substituents, including Br, Cl, CH_3_, NH_2_, CO_2_H and F, are used in the MIL-53 framework structure have also been explored in several studies (Ramsahye *et al.*, 2011 ▶; Couck *et al.*, 2012 ▶; Devic *et al.*, 2012 ▶; Biswas *et al.*, 2013 ▶) as well as the effect of other metals (Ga and In; Serra-Crespo *et al.*, 2012 ▶). The substituents investigated showed significant differences in the breathing behaviour under CO_2_ and hydrocarbon introduction, including strong intra-framework interactions that hold the framework constantly in the very narrow pore form, as well as changes in the phase transition behaviour, such as skipping the intermediate pore form upon gas uptake.

Similar breathing effects, in which transitions between narrow and large pores take place, have been studied crystallographically for a number of other MOFs, including: CO_2_ adsorption in MIL-47(V), where a breathing transition is observed along with a change from a monoclinic to ortho­rhombic unit cell (Leclerc *et al.*, 2011 ▶), N_2_ uptake in [Co(BDP)] (BDP = benzene-1,4-dipyrazolate) showing a dry (desolvated) phase, three distinct intermediate forms and a filled phase (Salles, Maurin *et al.*, 2010 ▶), CO_2_ adsorption in [Zn(BME-BDC)_2_(dabco)] (BME-BDC = 2,5-bis(2-methoxyethoxy)-1,4-benzenedicarboxylate) exhibiting narrow, intermediate and open pore forms (Henke *et al.*, 2011 ▶) and VO(BPDC) (BPDC = biphenyldicarboxylate) which exhibits a large and narrow pore form, but for which periodic DFT-D calculations predict an additional overstretched narrow pore form (Liu *et al.*, 2013 ▶).

*In situ* X-ray powder diffraction studies on [Co(HL^dc^)], [Cd(bpndc)(bpy)] and ZIF-8 have also shown evidence of gate-opening effects due to phase transitions in these flexible frameworks (Yang, Davies *et al.*, 2012 ▶; Tanaka *et al.*, 2008 ▶; Fairen-Jimenez *et al.*, 2011 ▶). The work on ZIF-8 (*aka* MAF-4) provides a good example of the relevance of such studies. The framework contains large cavities accessed through narrow windows which should provide a molecular sieving effect, denying access to larger gas molecules but allowing H_2_ in, yet experimental evidence demonstrates absorption of both CH_4_ and N_2_ (Fairen-Jimenez *et al.*, 2011 ▶). To address this problem Fairen-Jimenez *et al.* used *in situ* X-ray powder diffraction studies to show that the behaviour of the framework upon introduction of the gas was analogous to its behaviour under high physical pressures (*i.e.* 14.7 kbar in a diamond–anvil cell). The result was an enlargement of window size due to a swing effect involving the imidazolate rings, which allows the gas molecules to diffuse through the framework (Fairen-Jimenez *et al.*, 2011 ▶). This work was followed up by Zhang *et al.* (2012 ▶) who studied the effect by a combination of single-crystal X-ray diffraction and Raman spectroscopy. Although they showed the same two high- and low-pressure phases, they also found differences in the determined positions of the nitrogen molecules (Zhang *et al.*, 2012 ▶). Additionally they suggested the possibility of an intermediate phase, which comprised a solid solution of the geometries in the two identified phases.

**Figure 12:**
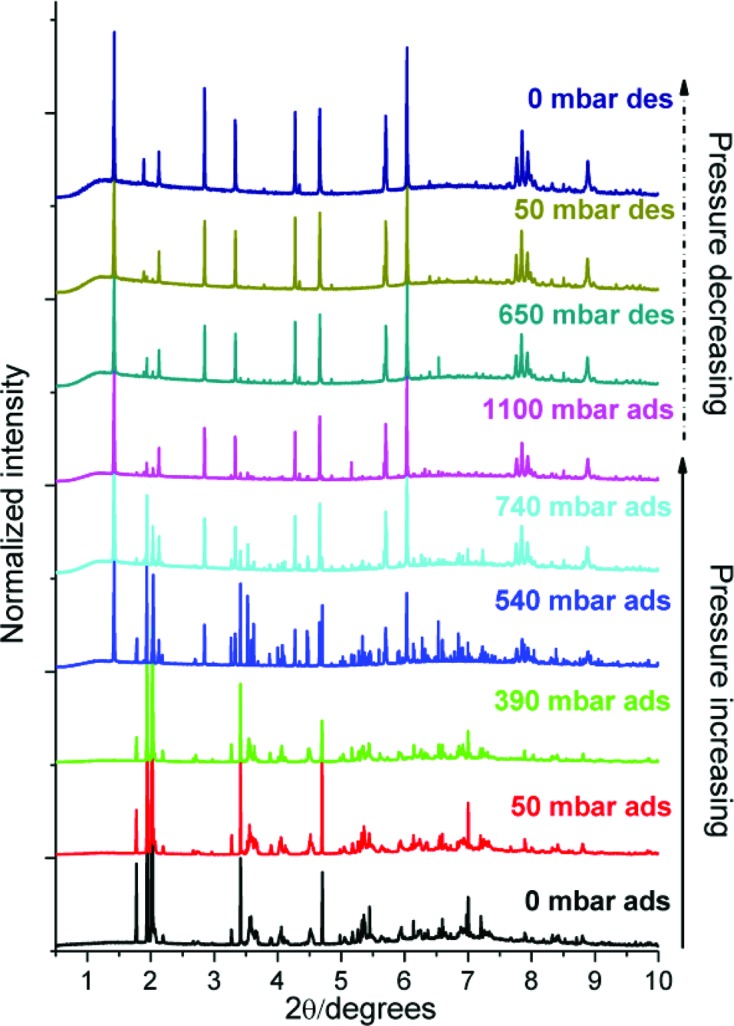
Powder diffraction patterns showing the irreversible structure changes that occur upon increased SO_2_ loading of NOTT-202a. (Reproduced from Yang *et al.*, 2013 ▶, http://dx.doi.org/10.1021/ja401061m, with permission from the Royal Society of Chemistry.)

Similar work by Schröder, Yang and co-workers on adsorption of SO_2_ in NOTT-202a (Me_2_NH_2_[In(L3)]) [H_4_L3 = biphenyl-3,3′,5,5′-tetra-(phenyl-4-carboxylic acid)] developed an interesting idea for accessing new MOF topologies. Adsorption of SO_2_ by the framework resulted in a phase transition which could be monitored by powder diffraction (Fig. 12 ▶). The introduction of the gas was not observed to break any bonds but just to cause a structural re-ordering that was maintained after removal of the gas (irreversible process). The unit cell of the new MOF (NOTT-202b) is related to the original structure and a model for the new architecture was obtained by analysis of the changes in symmetry. Unfortunately due to the possible disorder within the structure and insufficient data quality, Rietveld refinements were not successful, but the calculated powder pattern based upon the proposed structural model for NOTT-202b closely resembles the experimental pattern. The new MOF does not appear to be accessible by conventional synthetic means and therefore there may be a potential for using SO_2_ in a catalytic manner for MOF framework transformation (Yang *et al.*, 2013 ▶). Similarly, CO_2_ adsorption, coupled with the loss of coordinated solvent, has been implicated in the transformation of a three-dimensional MOF framework [Zn_4_(L5)_2_(DMF)_3_(OH_2_)_3_]·4H_2_O [H_4_L5 = 1,4-bis(3,5-dicarboxyphen-1-oxy)but-2-ene] to a two-dimensional framework [Zn_2_(L5)(OH_2_)_2_]·2H_2_O (Hawxwell *et al.*, 2007 ▶). The transformation involves a change in conformation of a flexible tetracarboxylate ligand (L5) from twisted to planar conformation. The structure of the resulting new MOF was determined *ab initio* by X-ray powder diffraction.

## 5. Functional groups   

Most MOFs employ linker ligands that contain only functional groups that bond to the metal ions in the framework. Such functional groups are usually carboxylate or azolate groups. Thus, the interior surfaces of most MOFs permit only relatively weak interactions with adsorbed guest molecules, including gases. A growing number of MOFs contain additional functional groups, either as part of secondary building units comprised of small metal-oxo/hydroxo clusters (*i.e.* as μ-OH or μ_3_-OH groups) or, increasingly, as substituents on the hydrocarbon backbone of the linker ligands. These functional groups provide the possibility of stronger and more directional host–guest interactions. Recent studies of gas adsorption in such functionalized MOFs have shown promise in either increased gas uptake over particular pressure ranges or increased selectivity for one gas over another due to favourable interactions between the framework and the gas molecules (Bourrelly *et al.*, 2005 ▶; Arstad *et al.*, 2008 ▶; Bae *et al.*, 2009 ▶; Couck *et al.*, 2009 ▶; Demessence *et al.*, 2009 ▶; Neofotistou *et al.*, 2009 ▶; Choi *et al.*, 2012 ▶). Crystallographic studies can reveal the role of the available functional groups within MOF pores in forming interactions with adsorbed gas molecules. This in turn may lead to the design of new MOFs with improved gas uptake characteristics.

**Figure 13:**
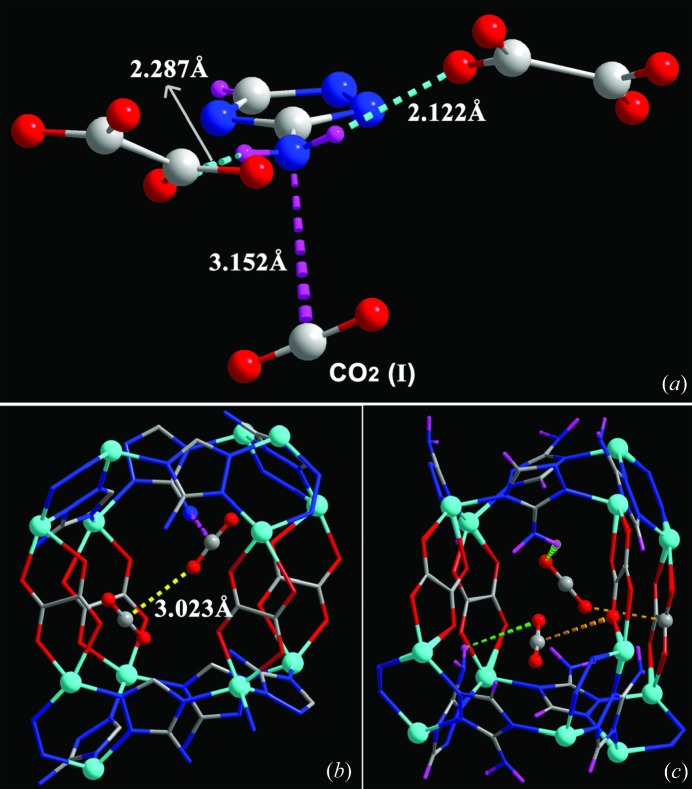
Representation of the different binding modes for CO_2_ molecules in [Zn_2_(Atz)_2_(ox)]·(CO_2_)_1.30_ at 173 K (Reproduced from Vaidhyanathan *et al.*, 2010 ▶, with permission from the AAAS.)

Shimizu and co-workers used *in situ* single-crystal X-ray diffraction to locate CO_2_ molecules inside [Zn_2_(Atz)_2_(ox)], a Zn-based MOF constructed using amino-functionalized 1,2,4-triazoles (Atz) and oxalate ligands (ox) (Vaidhyanathan *et al.*, 2009 ▶, 2010 ▶). The crystal structure was determined at four temperatures from 123 to 293 K. The best refinement (*R* = 0.027) was obtained at 173 K and indicated a loading of 1.3 CO_2_ per MOF formula unit, *i.e.* [Zn_2_(Atz)_2_(ox)](CO_2_)_1.30_, consistent with gravimetric adsorption measurements. Two independent CO_2_ molecules were located crystallographically inside the pore, with occupancies of 0.8 and 0.5 (Fig. 13 ▶). The molecule in site (I) forms an N^δ−^⋯C^δ+^ interaction between the N atom of the amino group and the C atom of a CO_2_ at a distance of 3.151 (8) Å (*cf.* Σ_vdW_ = 3.25 Å). The H atoms on the amine were located crystallographically, and noted to be bent out of the plane of the triazole, confirming that the amino lone pair was not delocalized into the ring and that therefore it could be responsible for the CO_2_ binding. Further, weak (long and highly non-linear) hydrogen bonds were identified between the amino groups and CO_2_ O atoms, as well as a characteristic cooperative T-shaped O^δ−^⋯C^δ+^ interaction between the two independent CO_2_ molecules.

Similar single-crystal diffraction studies on triazole-containing MOFs MAF-X7 and MAF-23 under CO_2_ loadings have also been carried out by Zhang and co-workers. The work on MAF-X7 showed one CO_2_ molecule within the framework that had a contact between the central carbon of the CO_2_ and the triazolate 4-nitrogen [3.26 (4) Å] at a separation close to the sum of their van der Waals radii. Additional, separate weak (long) C—H⋯O hydrogen bonds are noted involving the (DMF)H^+^ cation and a ring C—H group, each with CO_2_ O atoms (Lin *et al.*, 2011 ▶). In MAF-23 two independent CO_2_ sites were observed, both held in place by claw-like interactions from N atoms on two different triazolate rings. Most of the interaction distances were smaller than the sum of van der Waals radii and the site with a narrower claw angle showed a high CO_2_ occupancy (Liao *et al.*, 2012 ▶).

The N atoms of the amine or imine groups are not the only functional groups capable of affecting the adsorption of gases. Studies by Yang and Schröder using a combination of powder diffraction and inelastic neutron scattering has shown that the primary binding site for CO_2_ in NOTT-300 is a pocket containing a hydrogen bond from a μ_2_-OH group with an additional weak cooperative hydrogen bond from an aromatic C—H group (Yang, Sun *et al.*, 2012 ▶). The study also found a second CO_2_ molecule around the centre of the pore which interacted with the first CO_2_ (oxygen to carbon). Gravimetric measurements also suggested a high uptake of SO_2_ and crystallographic studies confirmed that the molecules sit in the same sites.

## 6. Open metal coordination sites   

**Figure 14:**
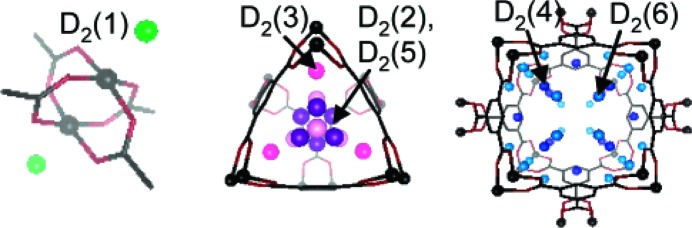
Six D_2_ adsorption sites identified in HKUST-1: the axial coordination site (left); view along the [111] direction showing sites close to the aromatic rings (sites 2 and 5) and carboxylate groups (site 3; middle); and view down the channels along the [100] direction (right). (Adapted from Peterson *et al.*, 2006 ▶, with permission from the American Chemical Society.)

Similar to the effect of unsaturated functional groups (see §5), MOFs with open metal coordination sites provide opportunities for direct interactions with gas molecules which may determine the location and even dominate the binding energy of the gas molecules. Several different frameworks with open metal coordination sites have been studied crystallographically under different gas environments. Work by Long and co-workers using neutron powder diffraction reported on the D_2_ binding sites in a MOF containing exposed Mn^2+^ ions (Dincă *et al.*, 2006 ▶). The work suggested two primary sites around the basic framework unit, with two further sites occupied at higher gas pressures. The first site reported was only 2.27 Å away from the exposed Mn^2+^ ion, suggesting a strong interaction from the ion. Despite this interaction, the occupancy of the site was only 29% due to competitive binding of methanol. Further work involved replacing the Mn^2+^ centres with Cu^2+^, which had a longer interaction with D_2_ of 2.47 Å, but resulted in increased occupancy of 93% due to more facile loss of the methanol (Dincă *et al.*, 2007 ▶). A contemporaneous study by Peterson *et al.* (2006 ▶) reported on six different D_2_ sites within the well studied MOF [Cu_3_(BTC)_2_] (HKUST-1) (Fig. 14 ▶). By refining the occupancies of the sites at different D_2_ loadings ranging from 0.5 D_2_ per Cu to 4 D_2_ per Cu they showed a progressive filling of the different adsorption sites within the framework. The main site was observed to be occupying the uncoordinated axial sites of the Cu paddlewheels at a distance of 2.39 (1) Å [*cf.* Cu—O 2.17 (1) Å in hydrated material]. Only at the highest loading were all six sites occupied, some still partially. Further work by the group in 2011 showed an additional three metastable sites at higher loadings of D_2_, up to 6.5 D_2_ per Cu (Peterson *et al.*, 2011 ▶), and illustrated the redistribution between sites that occurs on increasing gas loading. All experiments were carried out at 5 K after first loading the gas at higher temperatures.

The importance of open metal sites in HKUST-1 and also the limit of their involvement in interaction with gas molecules is exemplified in the neutron powder diffraction study of CD_4_ gas uptake reported by Kaskel and coworkers (Getzschmann *et al.*, 2010 ▶; Wu, Simmons, Liu *et al.*, 2010 ▶) and studies of noble gas uptake using powder diffraction with X-rays (Xe, Kr) and neutrons (Ar) by Forster and coworkers (Hulvey *et al.*, 2013 ▶). The crystallographic study by Kaskel identifies eight CD_4_ sites, four primary and four secondary sites, although not all can be occupied simultaneously due to the close proximity of some sites. Rietveld refinements for the evacuated MOF and at the two highest gas loadings (2.17 CD_4_/Cu and 3.67 CD_4_/Cu) show a reduction in Cu⋯Cu separation within the paddlewheel compared with the parent material with an axially coordinated water molecule. There is also a very small reduction in unit-cell volume upon gas uptake. The crystallographic study is complemented by a gravimetric gas adsorption study and a Grand Canonical Monte Carlo (GCMC) simulation of the adsorption at pressures and temperatures to match the experimental studies. The GCMC calculations provide a semi-quantitative model for the adsorption isotherms and identify methane sites that match most of those located crystallographically. However, the NPD study notably identifies a highly populated site that requires interaction with the open Cu sites (Cu⋯C 3.075 Å). The GCMC calculations are not parameterized to take into account this interaction with the metal centre and therefore do not identify this site. Zhou and Yildirim have studied the CO_2_ adsorption sites within HKUST-1 (Wu, Simmons, Srinivas *et al.*, 2010 ▶). The XRPD study showed two primary adsorption sites, one around the axial Cu coordination site and the other termed the ‘small cage window site’. The metal coordination site relates to the highest occupancy D_2_ site reported by Peterson *et al.* for HKUST-1 and the small cage window site is close to the corresponding second, third and sixth occupied sites (Peterson *et al.*, 2011 ▶). The two sites also resemble those observed for CD_4_ uptake in HKUST-1 in a similar study by Zhou and coworkers, which also examined CD_4_ uptake in other open-site Cu-MOFs experimentally and computationally (Wu, Simmons, Liu *et al.*, 2010 ▶). In contrast to these studies, investigation of noble gas uptake by Forster provided no evidence for interaction of the noble gas atoms with the open Cu^II^ sites (Hulvey *et al.*, 2013 ▶).

Very recently Matsuda, Kitagawa and coworkers have demonstrated the ability of a two-dimensional layered MOF, [Cu(aip)(OH_2_)]·*n*(solvent) (H_2_aip = 5-azidoisophthalic acid), which is based on the common *M*_2_(O_2_CR)_4_ paddlewheel motif, to undergo a transformation upon desolvation in which paddlewheel units from neighbouring layers become covalently linked into columns *via* bridging Cu—O bonds. This is a less extreme form of the reversible transformations between paddlewheels and metal carboxylate columns recently reported by us (Smart *et al.*, 2013 ▶) and by Bradshaw, Rossesinsky and coworkers (Stylianou *et al.*, 2012 ▶). The structural transformation is not only reversible upon resolvation, but provides a potential means for separation of CO and N_2_ gases. Study of the collapsed desolvated form [Cu(aip)] *via* simultaneous measurement of adsorption isotherms and X-ray powder diffraction at 120 K showed that initial adsorption of CO and N_2_ is analogous, involving filling of the larger of two channels, which accommodates 0.76 CO molecules (0.85 N_2_ molecules) per Cu. On increasing the pressure, there is a step change in the CO isotherm, but no such change in the N_2_ isotherm. Powder diffraction reveals a return of the framework containing CO to the two-dimensional layered arrangement of its solvated form, but in which CO molecules are axially coordinated at the Cu^II^ sites as well as filling both large and small channels (2.10 CO per Cu). Studies using CO/N_2_ gas mixtures demonstrate significant enrichment in CO upon adsorption by [Cu(aip)]. Although quite weakly coordinating, the results suggest that stronger coordination by CO than by N_2_ enables the structural transformation and therefore increased uptake of CO. Only upon expansion of the framework, following CO coordination, does the small channel become accessible, and in gas mixtures this is filled by CO rather than by N_2_, thereby accentuating the enrichment process. In a comparative study, the authors show that HKUST-1 exhibits no difference between adsorption isotherms of CO and N_2_, despite its well established ability to coordinate other adsorbed gas molecules at the open Cu^II^ sites (*see above*).

**Figure 15:**
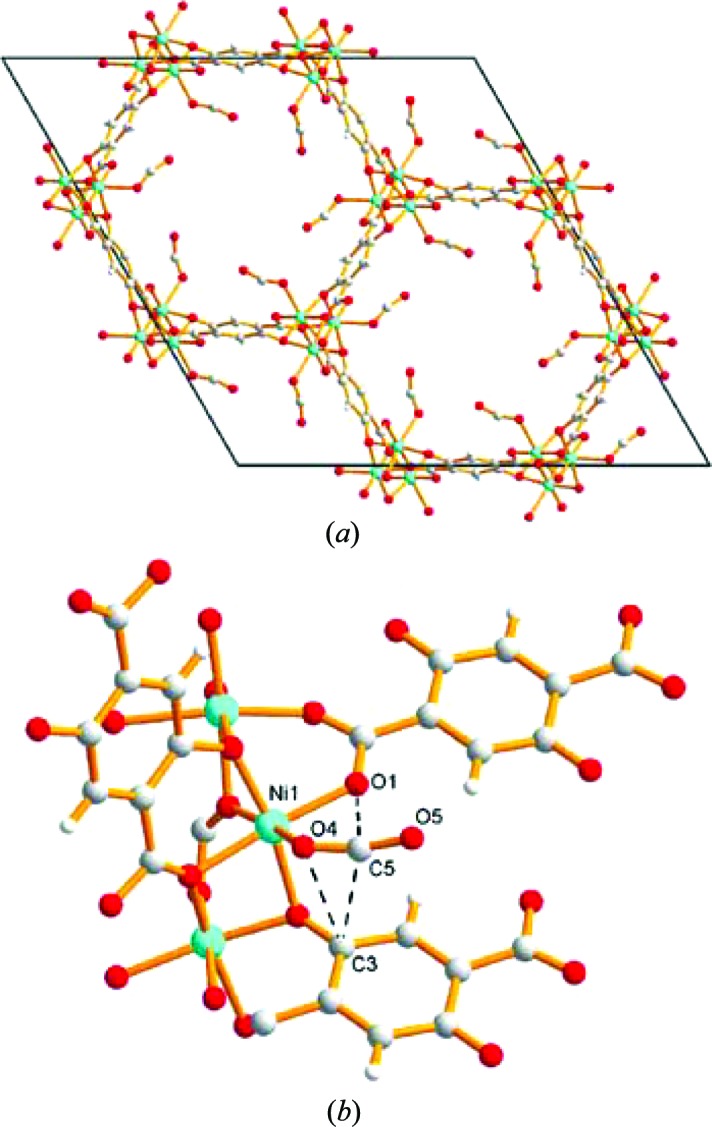
Crystal structure of CPO-27-Ni with adsorbed CO_2_ next to the metal atom. (*a*) Hexagonal channels viewed along the [001] direction. (*b*) Coordination environment of the CO_2_ molecules. (Reproduced from Dietzel *et al.*, 2008 ▶, http://dx.doi.org/10.1039/B810574J, with permission from the Royal Society of Chemistry.)

The channel-MOF CPO-27-Ni (Ni-MOF-74; Fig. 15 ▶) presents open Ni metal sites in the channel walls following desolvation. In 2008, both Morris and Blom reported *in situ* X-ray powder diffraction studies under loadings of NO (McKinlay *et al.*, 2008 ▶) and CO_2_ gas (Dietzel *et al.*, 2008 ▶), respectively. Both studies showed that the gas molecules were bound to the unsaturated Ni site with bond lengths shorter than the sum of the van der Waals radii. The NO gas bonded through the nitrogen and the carbon dioxide was bound end-on through an O atom but with a bent molecular arrangement (162°). In the same study Morris reported similar results for the cobalt analogue of the framework, which suggests that the effect is not specific to the metal and perhaps other less toxic metals could be targeted for applications such as *in vitro* release of NO. Along these lines it was suggested that the zinc analogue would be suitable, but unfortunately it proved considerably harder to activate for NO absorption (McKinlay *et al.*, 2008 ▶). A later study on CPO-27-Ni also showed applicability of the MOF to *in vitro* delivery of H_2_S, which similarly to the NO and CO_2_ studies was bound to the nickel centres, and was located using X-ray powder diffraction (Allan *et al.*, 2012 ▶).

The work on the CPO-27/MOF-74 series was further explored by Zhou and Yildirim who used neutron powder diffraction to investigate CO_2_ adsorption in the Mg analogue (Wu, Simmons, Srinivas *et al.*, 2010 ▶). The gas was adsorbed into the empty framework at 240 K and the sample cooled to 196 K, whereupon all gas had been absorbed. Further cooling to 20 K was undertaken prior to NPD data collection. The crystal structure model from Rietveld fitting (*R*_wp_ = 0.024) showed an end-on CO_2_ bound to the metal with a bent geometry (O=C=O ≃ 160.5°), which the authors attribute to effects of (unmodelled) disorder, since calculations suggest a significant energy penalty for such a large deviation from linearity. Indeed, a subsequent study by Brown and Yaghi, using NPD and MEM, suggested a larger O=C=O angle of 170° (Queen *et al.*, 2011 ▶). This study also found evidence of a second adsorption site within the pores that involved interaction of CO_2_ molecules with carbon and oxygen framework atoms.

Overall it can be seen that the open metal sites tend to have a significant effect on the location of a variety of different gases.

## 7. Gas sorption in molecular crystalline materials   

Although not formally the focus of this review it is pertinent to place the work on MOFs in a broader context and refer the reader to some examples of the growing number of crystallographic studies of gas and vapour sorption involving molecular crystals and coordination polymers. Such materials include flexible one-dimensional coordination polymers that adsorb a wide variety of gases (CO_2_, CH_4_, O_2_, H_2_, Ar, Kr, Xe) into small spaces between polymer strands (Takamizawa *et al.*, 2003 ▶, 2004 ▶, 2005 ▶; Takamizawa, Nakata & Akatsuka, 2006 ▶; Takamizawa, Kojima & Akatsuka, 2006 ▶; Takamizawa, Nakata, Akatsuka, Kachi-Terajima & Miyake, 2008 ▶, 2010 ▶; Takamizawa & Nakata, 2005 ▶; Ueda *et al.*, 2007 ▶; Kosaka, Yamagishi, Hori *et al.*, 2013 ▶; Kosaka, Yamagishi, Yoshida *et al.*, 2013 ▶;) or within cages (Coronado *et al.*, 2013 ▶) and both metal complexes and one-dimensional coordination polymers that adsorb gases or vapours *via* metal coordination: SO_2_ (Albrecht *et al.*, 2000 ▶); HCl/HBr (Mínguez Espallargas *et al.*, 2006 ▶, 2007 ▶, 2010 ▶, 2011 ▶; Adams *et al.*, 2007 ▶, 2010 ▶; Vitórica-Yrezábal *et al.*, 2011 ▶; Coronado *et al.*, 2012 ▶); N_2_/O_2_/H_2_/CO/C_2_H_4_/NH_3_ (Huang *et al.*, 2010 ▶); alcohol (*R*OH) vapours (Vitórica-Yrezábal *et al.*, 2013 ▶; Libri *et al.*, 2008 ▶). Organic molecular crystals which adsorb gases have been subject to crystallographic characterization for CO_2_ (Jacobs *et al.*, 2012 ▶, 2014 ▶) and Xe (Taratula *et al.*, 2010 ▶).

## 8. Conclusions   

Crystallographic studies in which adsorbed gas molecules are allocated within the pores of MOFs (or other porous materials) present significant experimental and structure refinement challenges. However, such studies have been successfully conducted on a range of metal–organic frameworks using both single-crystal and powder diffraction, and employing both X-rays and neutrons (Table 1 ▶). These studies have enabled important structural information on the position of gas molecules contained within these porous materials to be determined and the nature of the interactions involved in holding these molecules in place to be investigated. This knowledge can now be applied to help design the next generation of porous materials. As diffraction capabilities continue to advance, it is anticipated that crystallographic characterization of gas molecules adsorbed within MOFs and related porous materials will become more routinely undertaken. Such studies will continue to make important contributions not only to the development of MOFs and related materials, but in driving crystallography towards new frontiers. The latter is a most apt consideration at this time of the centenary of the field of crystallography.

## Figures and Tables

**Table 1:** Crystallographic studies of MOFs containing adsorbed gas molecules Abbreviations: NPD: neutron powder diffraction; PXRD: powder X-ray diffraction; SCND: single-crystal neutron diffraction; SCXRD: single-crystal X-ray diffraction; MEM: maximum entropy method; PDF: pair distribution function analysis; RT: room temperature. All abbreviations associated with the metal–organic framework names are provided in the *Appendix* [App appa].

MOF	Formula	Gas	Anaylsis method	Temperature (K)	Gas loading	Year	Reference
[Cd(bpndc)(bpy)]		O_2_	PXRD	100	0.8 bar	2008	Tanaka *et al.* (2008 ▶)
		Ar		110	3 bar		
		N_2_		90			
COMOC-2	[V(O)(BPDC)]	CO_2_	PXRD	233	0–17.5 bar	2013	Liu *et al.* (2013 ▶)
Co-BDP	[Co(BDP)]	N_2_	PXRD	100	0–15 bar	2010	Salles, Maurin *et al.* (2010 ▶)
[Co(HL^dc^)]		CO_2_	PXRD	195	0–1 bar	2012	Yang, Davies *et al.* (2012 ▶)
CPL-1	[Cu_2_(pzdc)_2_(pyz)]	O_2_	PXRD (Rietveld/MEM method)	300–90	0.8 bar	2002	Kitaura *et al.* (2002 ▶)
		N_2_				2005	Kitaura *et al.* (2005 ▶)
		Ar					
		CH_4_					
		C_2_H_2_		393–170	0.1 and 1.5 bar	2005	Matsuda *et al.* (2005 ▶), Kubota *et al.* (2006 ▶)
		H_2_		200–90	1.02 bar	2005	Kubota *et al.* (2005 ▶)
CPO-27-Ni (MOF-74)	[Ni_2_(dhtp)(OH_2_)_2_]	CO_2_	PXRD	100	0.2–0.5 atm	2008	Dietzel *et al.* (2008 ▶)
		H_2_S	PXRD	RT	1 atm	2012	Allan *et al.* (2012 ▶)
		NO	PXRD	298	1 atm	2008	McKinlay *et al.* (2008 ▶)
CPO-27-Co (MOF-74)	[Co_2_(dhtp)(OH_2_)_2_]						
CPO-27-Mg (MOF-74)	[Mg_2_(dhtp)(OH_2_)_2_]	CO_2_	NPD	20	0.64 CO_2_/Mg	2010	Wu, Simmons, Srinivas *et al.* (2010 ▶)
		CO_2_	NPD (Rietveld/MEM method)	20	0.5 and 1.75 CO_2_/Mg	2011	Queen *et al.* (2011 ▶)
[Cu(aip)]		CO_2_	PXRD	120	0–0.8 bar†	2014	Sato *et al.* (2014 ▶)
			PXRD (Rietveld/MEM method)	100	0.5 bar		
		N_2_	PXRD	120	0–0.8 bar†		
				77	0–1 bar†		
[Cu(pyrdc)(bpp)]_2_		CO_2_	SCXRD	193	Pressure unspecified: uptake 2 CO_2_/Cu	2005	Maji *et al.* (2005 ▶)
Cu-SIP-3	[Cu_2_(OH)(C_8_H_3_O_7_S)]	NO	SCXRD	RT	0.275–0.340 bar	2010	Allan *et al.* (2010 ▶)
DMOF	[Zn_2_(BDC)_2_(DABCO)]	CH_4_	SCXRD	90	Pressure unspecified: uptake 3.35 CH_4_/Zn	2009	Kim *et al.* (2009 ▶)
DMOF	[Zn_2_(BDC)_2_(DABCO)]·DSB	CO_2_	PXRD	195	0–0.8 bar	2011	Yanai *et al.* (2011 ▶)
		C_2_H_2_			0–0.6 bar		
DMOF-(BME)_2_	[Zn_2_(BME-BDC)_2_(DABCO)]	CO_2_	PXRD	195	0–1 bar	2011	Henke *et al.* (2011 ▶)
HKUST-1	[Cu_3_(BTC)_2_]	D_2_	NPD	5	2–6.5 D_2_/Cu	2006, 2011	Peterson *et al.* (2006 ▶, 2011 ▶)
		CD_4_	NPD	77	2.17–3.67 CD_4_/Cu	2010	Getzschmann *et al.* (2010 ▶)
		CD_4_	NPD	4	1.1 CD_4_/Cu	2010	Wu, Simmons, Liu *et al.* (2010 ▶)
		CO_2_	NPD	20	1.07–1.47 CO_2_/Cu	2010	Wu, Simmons, Srinivas *et al.* (2010 ▶)
		Ar	NPD	8	0.17 (3) Ar/Cu	2013	Hulvey *et al.* (2013 ▶)
		Kr	PXRD	140–200	0.075 (1)–0.374 (4) Kr/Cu (3 loadings)	2013	Hulvey *et al.* (2013 ▶)
		Xe	PXRD	240–260	0.072 (1)–0.315 (2) Xe/Cu (5 loadings)	2013	Hulvey *et al.* (2013 ▶)
HCu[(Cu_4_Cl)_3_(BTT)_8_]·3HCl		D_2_	NPD	4	6–30 D_2_/formula unit (13 Cu)	2007	Dincă *et al.* (2007 ▶)
Mn_3_[(Mn_4_Cl)_3_(BTT)_8_(CH_3_OH)_10_]_2_		D_2_	NPD	3.5	12 D_2_/formula unit (27 Mn)	2006	Dincă *et al.* (2006 ▶)
[Mg(O_2_CH)_2_]		C_2_H_2_	SCXRD	90	Pressure unspecified: uptake 0.33 C_2_H_2_/Mg (or Mn)	2007	Samsonenko *et al.* (2007 ▶)
[Mn(O_2_CH)_2_]							
MAF-2	[Cu(etz)]	N_2_	SCXRD	93	Pressure unspecified: uptake‡ 1.0 N_2_/Cu	2008	Zhang & Chen (2008 ▶)
		C_2_H_2_		293	10–20 atm: uptake 1 C_2_H_2_/Cu	2009	Zhang & Chen (2009 ▶)
		C_2_H_2_		123	≤ 0.8 bar: uptake 1 CO_2_ or C_2_H_2_/Cu		
		CO_2_					
		C_2_H_2_		195	0.05–0.8 bar: uptake 0.04–0.42 CO_2_ or C_2_H_2_/Cu		
		CO_2_					
ZIF-8 (MAF-4)	[Zn(MeIm)_2_]	D_2_	NPD	3.5	0.5–4.67 D_2_/Zn	2007	Wu *et al.* (2007 ▶)
		CD_4_	NPD	100–3.5	1–3 CD_4_/Zn	2009	Wu *et al.* (2009 ▶)
		N_2_	PXRD	77	0.4 bar	2011	Fairen-Jimenez *et al.* (2011 ▶)
		N_2_	SCXRD	423–100	Open-flow N_2_ cryostat	2012	Zhang *et al.* (2012 ▶)
MAF-23	[Zn_2_(BTM)_2_]	CO_2_	SCXRD	195	0–1.5 CO_2_/Zn	2012	Liao *et al.* (2012 ▶)
MAF-X7	(Me_2_NH_2_)(Hdmf) [Co_2_Cl_4_(ppt)_2_]	CO_2_	SCXRD	120	Sealed at 1 atm at 273 K	2011	Lin *et al.* (2011 ▶)
MCF-27	[LiZn(BTC)]	CO_2_	SCXRD	195	Unspecified	2010	Xie *et al.* (2010 ▶)
		N_2_		103			
MFU-4*l*	[Zn_5_Cl_4_(BTDD)_3_]	Xe	PXRD (Rietveld/MEM method)	110–150	0.02 bar at RT	2012	Soleimani-Dorcheh *et al.* (2012 ▶)
MIL-47(V)	[V(O)(BDC)]	CH_4_	PXRD	200	0–8.84 bar	2010	Rosenbach *et al.* (2010 ▶)
		C_3_H_8_		303	0–8.28 bar		
		CO_2_	PXRD	303	0–30.9 bar	2011	Leclerc *et al.* (2011 ▶)
		CO_2_	PXRD	200	0–1.53 bar	2010	Salles, Jobic *et al.* (2010 ▶)
MIL-53(Cr) (hydrated)	[Cr(OH)(BDC)]·*x*H_2_O	CO_2_	PXRD	Unspecified§	1–15 bar	2006	Llewellyn *et al.* (2006 ▶)
MIL-53(Cr)	[Cr(OH)(BDC)]	CO_2_	PXRD	293	0–10 bar	2007	Serre *et al.* (2007 ▶)
				195	1 bar		
		CH_4_	PXRD	303	0–33 bar	2008	Llewellyn *et al.* (2008 ▶)
		C_2_H_6_			0–13.5 bar		
		C_3_H_8_			0–10 bar		
		C_4_H_10_			0–0.5 bar		
		CO_2_/CH_4_ mixture	PXRD	303	0–30 bar (25:75, 50:50, 75:25)	2009	Hamon *et al.* (2009 ▶)
		H_2_	PXRD	303	0–30 bar	2009	Salles *et al.* (2009 ▶)
MIL-53(Fe)	[Fe(OH)(BDC)]	CH_4_	PXRD	303	0–43 bar	2009	Llewellyn *et al.* (2009 ▶)
		C_2_H_6_			0–37 bar		
		C_3_H_8_			0–8.6 bar		
		C_4_H_10_			0–2.1 bar		
		CO_2_		230	0–8.8 bar	2012	Devic *et al.* (2012 ▶)
	[Fe(OH)(BDC-Cl)]	CH_4_	PXRD	303	0–38.7 bar	2011	Ramsahye *et al.* (2011 ▶)
		C_2_H_6_			0–36.3 bar		
		C_3_H_8_			0–8 bar		
		C_4_H_10_			0–2.1 bar		
		CO_2_		230	0–9.8 bar	2012	Devic *et al.* (2012 ▶)
	[Fe(OH)(BDC-Br)]	CH_4_	PXRD	303	0–40.3 bar	2011	Ramsahye *et al.* (2011 ▶)
		C_2_H_6_			0–37.7 bar		
		C_3_H_8_			0–8.2 bar		
		C_4_H_10_		303–263	0–0.7 bar		
		CO_2_		230	0–10.7 bar	2012	Devic *et al.* (2012 ▶)
	[Fe(OH)(BDC-CH_3_)]	CH_4_	PXRD	303	0–42.0 bar	2011	Ramsahye *et al.* (2011 ▶)
		C_2_H_6_			0–38.2 bar		
		C_3_H_8_			0–1.2 bar		
		CO_2_		230	0–10 bar	2012	Devic *et al.* (2012 ▶)
	[Fe(OH)(BDC-NH_2_)]	CO_2_	PXRD	230	0–11.9 bar	2012	
	[Fe(OH)(BDC-CO_2_H)]	CO_2_		230	0–11 bar	2012	
MIL-53(Al)	[Al(OH)(BDC-NH_2_)]	CO_2_	PXRD	253	0–18 bar	2012	Couck *et al.* (2012 ▶), Serra-Crespo *et al.* (2012 ▶)
		CH_4_			0–15 bar		
	[Al(OH)(BDC-F)]	CO_2_	PXRD	233–193	0–1.47 bar	2013	Biswas *et al.* (2013 ▶)
MIL-53(Ga)	[Ga(OH)(BDC-NH_2_)]	CO_2_	PXRD	253	0–16 bar	2012	Serra-Crespo *et al.* (2012 ▶)
	[Ga(OH)(BDC-NH_2_)]	CH_4_			0–11 bar		
MIL-53(In)	[In(OH)(BDC-NH_2_)]	CO_2_	PXRD	253	0–20 bar		
	[In(OH)(BDC-NH_2_)]	CH_4_			0–14 bar		
MIL-53(Sc)	[Sc(OH)(BDC)]	CO_2_	PXRD	196	0–0.9 bar	2013	Chen *et al.* (2013 ▶)
MIL-88B(Fe)	[Fe(OH)(BDC-NO_2_)]	NO	PXRD	RT	1 bar	2013	McKinlay *et al.* (2013 ▶)
	[Fe(OH)(BDC-2OH)]	NO	PXRD	RT	1 bar	2013	McKinlay *et al.* (2013 ▶)
MOF-5	[Zn_4_O(BDC)_3_]	H_2_	SCND	300–5	1 atm	2006	Spencer *et al.* (2006 ▶)
		D_2_	NPD	3.5	1–11.5 D_2_/Zn	2005	Yildirim & Hartman (2005 ▶)
		He	PXRD	100–500	1.7–150 bar	2013	Lock *et al.* (2013 ▶)
		CD_4_	NPD	100–3.5	0.75–6 CD_4_/Zn	2009	Wu *et al.* (2009 ▶)
		N_2_	SCXRD	293–30	1.25–2.5 N_2_ or Ar/Zn	2005	Rowsell, Spencer *et al.* (2005 ▶)
		Ar					
NOTT-202a¶	(Me_2_NH_2_)[In(L3)]	CO_2_	PXRD	195, 273	0–1 bar	2012	Yang, Lin *et al.* (2012 ▶)
		SO_2_		273	0–1.1 bar	2013	Yang *et al.* (2013 ▶)
NOTT-300	[Al_2_(OH)_2_(L4)]	CO_2_	PXRD	273	0–1 bar	2012	Yang, Sun *et al.* (2012 ▶)
		SO_2_					
PCN-11	[Cu_2_(sbtc)]	CD_4_	NPD	4	2.8 CD_4_/Cu	2010	Wu, Simmons, Liu *et al.* (2010 ▶)
[Sc_2_(BDC)_3_]		CO_2_	SCXRD	235	1 bar	2009	Miller *et al.* (2009 ▶)
		H_2_		80	0.25 bar		
		CH_4_		230	9 bar		
		C_2_H_6_		230	5 bar		
SNU-110	[Zn_2_(mpm-PBODB)_2_(bpy)]	CO_2_	PXRD	248	1 atm (CO_2_ stream)	2012	Hong & Suh (2012 ▶)
YO-MOF	[Zn_2_(L1)(L2)]	CO_2_	PXRD–PDF analysis	260–RT	1 atm	2010	Mulfort *et al.* (2010 ▶)
Y(BTC)		D_2_	NPD	4	0.64 (5)–5.53 (3) D_2_/Y (6 loadings)	2008	Luo *et al.* (2008 ▶)
[Zn_2_(Atz)_2_(ox)]		CO_2_	SCXRD	123–293	0.65 CO_2_/Zn	2010	Vaidhyanathan *et al.* (2010 ▶)
[Zn_2_(btdc)_2_(bpy)] (threefold interpenetrated)		CO_2_	PXRD	195	0–1 bar†	2010	Bureekaew *et al.* (2010 ▶)
[Zn_2_(btdc)_2_(bpy)] (twofold interpenetrated)							
[Zn_2_(sdb)_2_(bpy)]		CO_2_	PXRD	195–295	0.1–0.9 bar	2013	Hijikata *et al.* (2013 ▶)
[Zn(TCNQ-TCNQ)(bpy)]		O_2_	PXRD	Unspecified	7.5 O_2_/Zn	2010	Shimomura *et al.* (2010 ▶)
		NO			9 NO/Zn		

† Simultaneous measurement of adsorption isotherm and X-ray powder pattern.

‡ The same study is cited by the same authors in a later report (Zhang & Chen, 2009 ▶), but listed as having an uptake of 0.5 N_2_/Cu (*i.e.* formula MAF-2·0.5N_2_).

§ Accompanying adsorption isotherms over similar pressure range are conducted at 304 K.

¶ NOTT-202a is the desolvated form of NOTT-202, which has the formula (Me_2_NH_2_)_1.75_[In(L3)]_1.75_·12DMF·10H_2_O (Yang, Lin *et al.*, 2012 ▶).

## Appendix A Abbreviations

H_2_aip: 5-azidoisophthalic acid

Atz: 3-amino-1,2,4-triazole

BDC: 1,4-benzenedicarboxylate

BDP: 1,4-benzenedipyrazolate

BME-BDC: 2,5-bis(2-methoxyethoxy)-1,4-benzenedicarboxy­late

BPDC: biphenyl-4,4′-dicarboxylate

H_2_bpndc: benzophenone-4,4′-dicarboxylic acid

bpy: 4,4′-bipyridyl

BTC: 1,3,5-benzenetricarboxylate

H_2_btdc: 2,2′-bithiophene-5,5′-dicarboxylic acid

H_2_BTDD: bis(1*H*-1,2,3-triazolo[4,5-*b*],[4′,5′-*i*])dibenzo[1,4]dioxin

H_3_BTT: 1,3,5-tris(tetrazol-5-yl)benzene

DABCO: 1,4-diazabicyclo[2.2.2]octane

DMF: dimethylformamide

DSB: distyrylbenzene

Hetz: 3,5-diethyl-1,2,4-triazole

H_4_L1: 4,4′,4′′,4′′′-benzene-1,2,4,5-tetrayl-tetrabenzoic acid

L2: *N*,*N*′-di-(4-pyridyl)-1,4,5,8-naphthalenetetracarboxydiimide

H_4_L3 (NOTT-202a): biphenyl-3,3′,5,5′-tetra-(phenyl-4-carboxylic acid)

H_4_L4 (NOTT-300): biphenyl-3,3′,5,5′-tetracarboxylic acid

H_4_L5: 1,4-bis(3,5-dicarboxyphen-1-oxy)but-2-ene

H_3_L^dc^: 5-{4-[3-carboxy-2,6-bis(pyridin-4-yl)pyridin-4-yl]phenyl}benzene-1,3-dicarboxylic acid

MeIm: 2-methylimidazolate

H_2_mpm-PBODB: 3,3′-(1,4-phenylenebis(oxy))dibenzoic acid

ox: oxalate

H_2_ppt: 3-(2-phenol)-5-(4-pyridyl)-1,2,4-triazole

pyz: pyrazine

pzdc: 2,3-pyrazinedicarboxylate

—*R*: (—CH_3_) [with additional group (methyl) instead of a H atom]

H_2_sdb: 4,4-sulfonyldibenzoic acid

H_2_sbtc: *trans*-stilbene-3,3′,5,5′-tetracarboxylic acid

TCNQ: 7,7,8,8-tetracyano-*p*-quino-dimethane
